# Spirituality is associated with immune parameters and disease activity in primary Sjögren’s syndrome: a cross-sectional study

**DOI:** 10.1038/s41598-024-62801-w

**Published:** 2024-05-30

**Authors:** László V. Módis, Zsófia Aradi, Ildikó Fanny Horváth, Péter Pikó, Gábor Papp, Mátyás Osváth, Antónia Szántó, Antal Bugán

**Affiliations:** 1 Faculty of Medicine, Department of Behavioural Sciences, University of Debrecen, Debrecen, 4032 Hungary; 2 Szabolcs-Szatmár-Bereg County Teaching Hospital, Nagykálló Sántha Kálmán Member Hospital, Nagykálló, 4320 Hungary; 3 Faculty of Medicine, Department of Internal Medicine, Division of Clinical Immunology, University of Debrecen, Debrecen, 4032 Hungary; 4 Faculty of Medicine, Department of Public Health and Epidemiology, University of Debrecen, Debrecen, 4028 Hungary; 5 National Laboratory for Health Security, Center for Epidemiology and Surveillance, Semmelweis University, Budapest, 1089 Hungary; 6 PractiWork Plc, Budapest, 1037 Hungary

**Keywords:** Quality of life, Rheumatic diseases

## Abstract

The role of spirituality in health and disease is a complex and emerging area of research. Incorporating spirituality into the bio-psycho-social model of health and disease leading to the bio-psycho-social-spiritual model provides a more comprehensive framework. In this context, chronic disorders like primary Sjögren’s syndrome (pSS) are of interest due to their intricate interactions between biological, psychological, and spiritual factors. This study explored the relationship between spirituality, immune parameters, and disease activity in pSS patients. Data from 108 patients were analyzed, including self-assessed spirituality (answering to direct questions and completing the Spiritual Transcendence Scale), immunological parameters and disease activity scores. The findings revealed several associations. Individuals with spiritual attitudes or engaged in regular prayer/meditation showed lower serum levels of autoantibodies specific to pSS and lower disease activity scores. Spiritual engagement was also linked to decreased perceived skin and tracheal dryness, suggesting potential benefits for physical symptoms. These findings suggest that spirituality may play a significant role in modulating immune responses and disease activity in pSS patients. The study underscores the importance of considering spirituality as an integral part of the holistic approach to health and disease, further expanding the understanding of the interconnectedness of biological, psychological, and spiritual dimensions.

## Introduction

Factors determining health and disease are framed within the bio-psycho-social (BPS) model of health^1^. Components of this model form a complex system with circular causality, representing health as a dynamic, sensitive and volatile balance that can be easily disrupted resulting in disease. In recent decades many papers have been published reporting that besides the elements of BPS framework, spirituality plays an important role in maintaining health balance and in the pathways leading to different diseases^2–5^. Although spirituality is a fluid concept with uncertainty in its definition and exact meaning, there are mutual points among the different approaches. Spirituality is universal to all mankind, its essence is to search for, (or unite with) the transcendent, the sacred or the numinous and it has a motivating effect encouraging one to seek for purpose or meaning of life^6^. It is also quite consensual, that spirituality has biological correlates^7^. Spirituality has attracted the interest of the scientific community engaged in medicine and health psychology turning out to be a significant determinant of health and disease. Therefore, there is an emerging opinion in the literature declaring that the BPS model should be expanded to include the spiritual dimension, resulting in the bio-psycho-social-spiritual (BPSS) model^8–10^. In correspondence to this concept, in the case of systemic autoimmune disorders, alongside with biological causes and symptoms, spirituality has also been observed as an independent variable determining the characteristics of the disease. In rheumatoid arthritis (RA) spirituality was established as an independent predictor of happiness and positive health perceptions and facilitated emotional adjustment and resilience among patients^11^. Also, among RA patients, spirituality was described as a beneficial factor in the posttraumatic growth, which is a positive psychological response to the disease^12^. A more recent Turkish study found a negative correlation between psychological and spiritual well-being in RA^13^. According to a case report, pastoral care and religious support, applied alongside with pharmacological treatment, had some beneficial effects on anxiety and depressive mood, but no significant effect on patient’s physical functions in osteoarthritis^14^. Strong family relationships and spiritual beliefs were justified as major coping strategies in systemic lupus erythematosus^15^. In correspondence with the BPS model, many psychological aspects have been described in primary Sjögren’s syndrome (pSS), such as depression and anxiety^16,17^ and typical personality patterns^16,18–20^. However, no studies have been published about the role of the spiritual dimension in this disease. Considering the importance of spirituality in other systemic autoimmune diseases described above, it is likely that it plays a role in the pathogenesis and disease course of pSS. Furthermore, the correlation between pSS and personality also underlines this hypothesis, since spirituality appears as a factor of personality in modern theories^21,22^. In line with all this, the aim of this study was to establish the role of spirituality in pSS and to examine its interaction with immune parameters and disease activity in pSS patients. Since religion is an overlapping phenomenon examined usually alongside with spirituality, we also considered religious beliefs of the patients.

## Results

### Comparison of immune parameters and disease activity scores between non-spiritual and spiritual groups

First, we established a comparison between sociodemographic characteristics, self-assessed measures of spirituality and religiousness between non-spiritual and spiritual groups (see grouping perspectives at the "Methods" section). There were no significant differences regarding disease duration, percentage of females, settlement type, education, living in partnership and smoking between the two groups. Significant alterations were detected regarding age (62.23 vs. 57.40; p = 0.028) and spiritual/religious features expectedly (Table 1).

**Table 1:** Comparison of the sociodemographic characteristics and self-assessed measures of spirituality and religiousness of the research sample between non-spiritual and spiritual groups.

		Non-spiritual (n = 48)	Spiritual (n = 60)	p-value
		Average (95%CI)		
Age (year)		62.23 (58.75–65.71)	57.40 (54.28–60.52)	0.028*
Disease duration		15.91 (13.19–18.64)	14.95 (12.64–17.26)	0.653
Duration of prayer/meditation (hours/week)		0.39 (0.00–0.82)	1.12 (0.59–1.65)	< 0.001**
		Prevalence in % (95% CI)		p-value
Female		91.7 (81.4–97.1)	88.3 (78.5–94.6)	0.569
Settlement type	Capital or city	41.7 (28.5–55.8)	35.0 (23.9–47.5)	0.632
	Small town	37.5 (24.9–51.6)	36.7 (25.3–49.3)	
	Rural	20.8 (11.2–33.8)	28.3 (18.1–40.6)	
Education	Not completed or completed primary school	10.4 (4.1–21.3)	6.7 (2.3–15.1)	0.402
	High school	58.3 (44.2–71.5)	50.0 (37.6–62.4)	
	College/university diploma	31.3 (19.5–45.2)	43.3 (31.4 – 55.9)	
Living in partnership		64.6 (50.5–76.9)	65.0 (52.5 – 76.1)	0.964
Smokers		6.3 (1.8–15.7)	5.0 (1.4 – 12.7)	0.778
Engagement in prayer/meditation		16.67 (8.21–29.00)	48.33 (36.02 – 60.81)	< 0.001**
Self-assessed level of religiousness	Not or little religious	72.9 (59.3–83.9)	33.3 (22.4–45.8)	< 0.001**
	Religious or very religious	27.1 (16.1–40.7)	66.7 (54.2–77.6)	

Confidence interval level of 95% was applied.

*CI* confidence interval.

*p < 0.05; **p < 0.01.

Semi-objective disease activity score ESSDAI was significantly lower in the spiritual group comparing to the non-spiritual one (2.18 vs. 2.88; p = 0.010). The same difference was noted regarding the intensity of perceived vaginal dryness (2.13 vs. 3.51; p = 0.041). However, no significant differences were found between IGG, RF, autoantibodies SSA and SSB, ESSPRI score and ESSPRI items 1–9 between the two groups. (Table 2).

**Table 2:** Comparison of immune parameters and disease activity measures between non-spiritual and spiritual subgroups.

	Non-spiritual (n = 48)	Spiritual (n = 60)	p-value
	Average (95%CI)		
IGG	14.45 (12.54–16.35)	14.58 (12.28–6.88)	0.654
RF	49.90 (14.24–85.55)	30.57 (16.01–45.12)	0.371
SSA	46.92 (36.41–57.42)	42.43 (33.47–51.40)	0.559
SSB	36.48 (25.89–47.07)	27.05 (19.24–34.86)	0.289
ESSDAI	2.88 (1.97–3.78)	2.18 (1.10–3.27)	0.010*
ESSPRI score	5.35 (4.74–5.95)	4.97 (4.30–5.64)	0.445
ESSPRI main items (1–3)			
dryness (ESSPRI01)	5.46 (4.82–6.10)	5.29 (4.66–5.92)	0.847
fatigue (ESSPRI02)	5.67 (4.92–6.41)	4.93 (4.17–5.69)	0.186
limb pain (ESSPRI03)	4.92 (4.04–5.79)	5.03 (4.19–5.88)	0.902
ESSPRI items 4–10			
mental fatigue (ESSPRI04)	2.64 (1.99–3.28)	3.20 (2.52–3.89)	0.348
ESSPRI dryness items			
ocular (ESSPRI05)	5.28 (4.52–6.04)	5.03 (4.37–5.69)	0.564
oral (ESSPRI06)	4.89 (4.05–5.74)	5.54 (4.84–6.24)	0.233
skin (ESSPRI07)	5.06 (4.39–5.74)	4.63 (3.88–5.38)	0.398
nasal (ESSPRI08)	3.46 (2.61–4.31)	3.71 (2.96–4.46)	0.634
tracheal (ESSPRI09)	3.70 (2.86–4.55)	3.98 (3.20–4.77)	0.633
vaginal (ESSPRI10)	3.51 (2.54–4.49)	2.13 (1.41–2.85)	0.041*

Confidence interval level of 95% was applied.

*IGG* immunoglobulin G, *RF* rheumatoid factor, *SSA* anti-Ro/SSA autoantibody, *SSB* anti-La/SSB autoantibody, *ESSDAI* EULAR Sjögren’s syndrome disease activity index, *ESSPRI* EULAR Sjögren’s syndrome patient reported index (ESSPRI), *CI* confidence interval.

*p < 0.05; **p < 0.01.

The analysis was also run between the same groups regarding blood cell counts NEU and LY, HGB, PLT and complement activities, resulting in no significant alterations (Supplementary Table 2). Group comparison was performed also between not or little religious and religious or very religious groups. Lymphocyte count was higher in the religious or very religious group (1.69 vs. 1.39; p = 0.019), while perceived dryness was decreased in the same group (4.81 vs. 5.89; p = 0.035). The other examined biological and disease activity markers did not show any significant change (Supplementary Table 3).

### Linear relations of spirituality, engagement in prayer/meditation and its duration and immune and disease activity parameters

**Figure 1:**
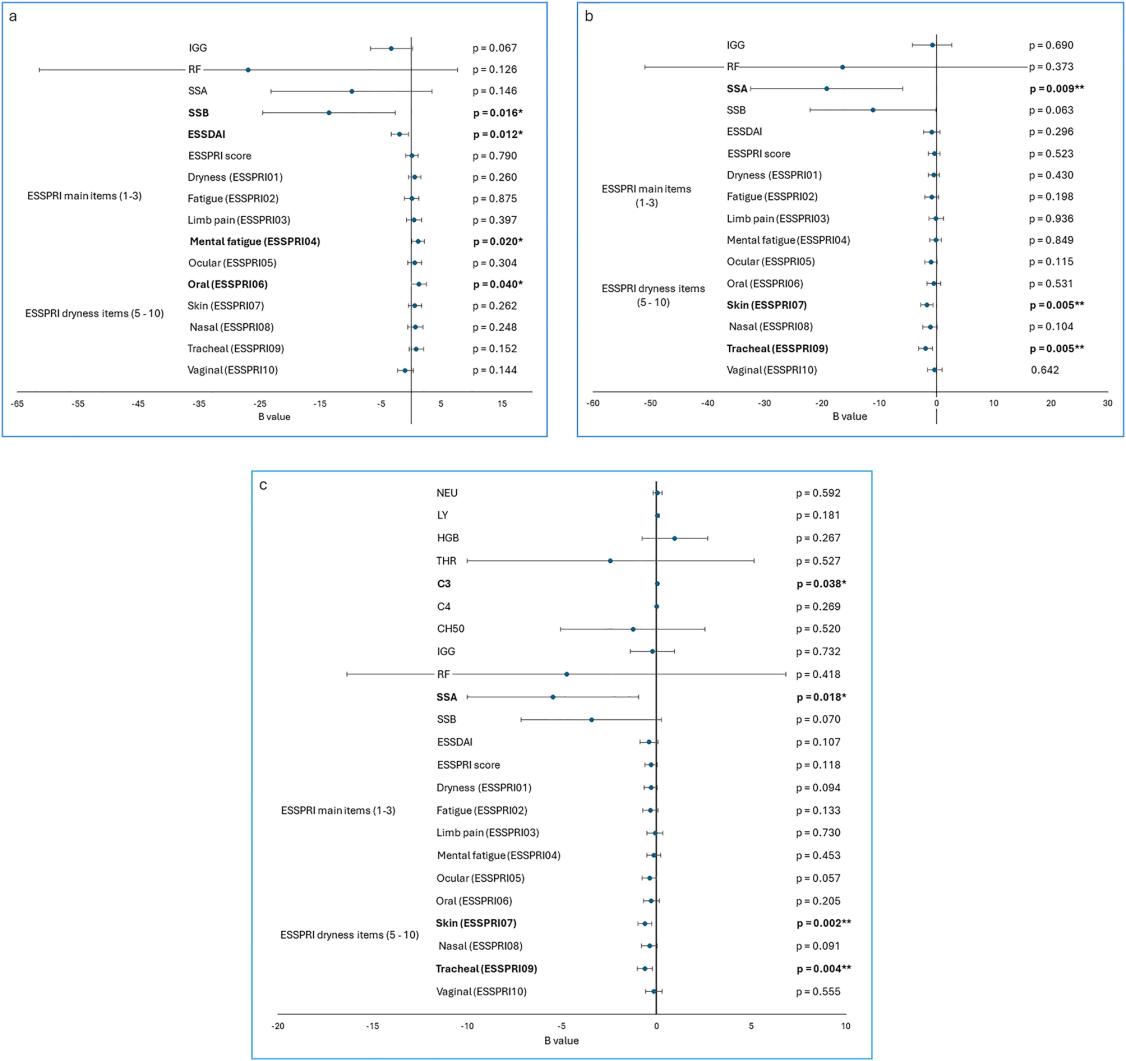
Linear regression adjusted for sex, age, disease duration, settlement type, education, living in partnership, smoking, religiousness (**a**) the effect of spirituality on disease activity markers, adjusted for engagement in and duration of prayer/meditation in addition to the factors mentioned above. (**b**) the effect of engagement in prayer/meditation on disease activity markers, adjusted for spirituality and duration of prayer/meditation in addition to the factors mentioned above. (**c**) the effect of duration of prayer/meditation on disease activity markers, adjusted for spirituality and engagement in prayer/meditation in addition to the factors mentioned above. *IGG* Immunoglobulin G, *RF* rheumatoid factor, *SSA* anti-Ro/SSA autoantibody, *SSB* anti-La/SSB autoantibody, *ESSDAI* EULAR Sjögren’s Syndrome Disease Activity Index, *ESSPRI* EULAR Sjögren’s Syndrome Patient Reported Index (ESSPRI). *p < 0.05; **p < 0.01.

Linear regression analyses adjusted for sex, age, disease duration, settlement type, education, living in partnership, smoking, religiousness, spirituality, and engagement in prayer/meditation were run to establish linear relations between spirituality and immune/disease activity parameters. High level of spirituality supposed lower SSB serum level (B = − 13.495; p = 0.016), lower ESSDAI score (B = − 1.859; p = 0.012) and enhanced perceived mental fatigue (B = 1.222; p = 0.020) and oral dryness (B = 1.293; p = 0.04. Regarding IGG, negative correlation with spirituality at borderline significance was detected (B = − 3.324; p = 0.067). RF, SSA and ESSPRI score was not significantly linked to spirituality (Fig. 1a). Furthermore, engagement in prayer/meditation predicted reduced SSA level (B = − 16.414; p = 0.009) and perceived skin and tracheal dryness (B = − 1.682; p = 0.005; B = − 1.852; p = 0.005, respectively) (Fig. 1b). Religiousness was associated with elevated IGG (B = 3.732; p = 0.045), SSA (B = 15.731; p = 0.027) and SSB level (B = 14.744; p = 0.012) (Supplementary Table 4). Duration of individual spiritual activity was associated with the same factors as engagement in it itself (SSA (B = − 5.458; p = 0.018), perceived skin and tracheal dryness (B = − 0.595; p = 0.020; B = − 0605; p = 0.040, respectively)). In addition, positive association with complement component C3 activity was observed (B = 0.043; p = 0.038) (Fig. 1c).

The same analysis was performed in the case of STS subscales with few significant results. Prayer/Meditation Enjoyment was negatively linked to perceived vaginal dryness (B = -0.118; p = 0.038). Universal Connectedness and Greater Purpose were detected to be negatively related to complement component C3 activity (B = − 0.020; p = 0,003; B = − 0.026; p = 0.026, respectively). Higher levels on Closeness to the Deceased subscale resulted in elevated perceived mental health (B = 0.276; p = 0.014). Other significant results were not discovered on any subscales (Supplementary Table 5).

### Logistic relations of religiousness, spirituality, engagement in prayer/meditation and its duration and ESSDAI, ESSPRI, SSA and SSB

**Figure 2:**
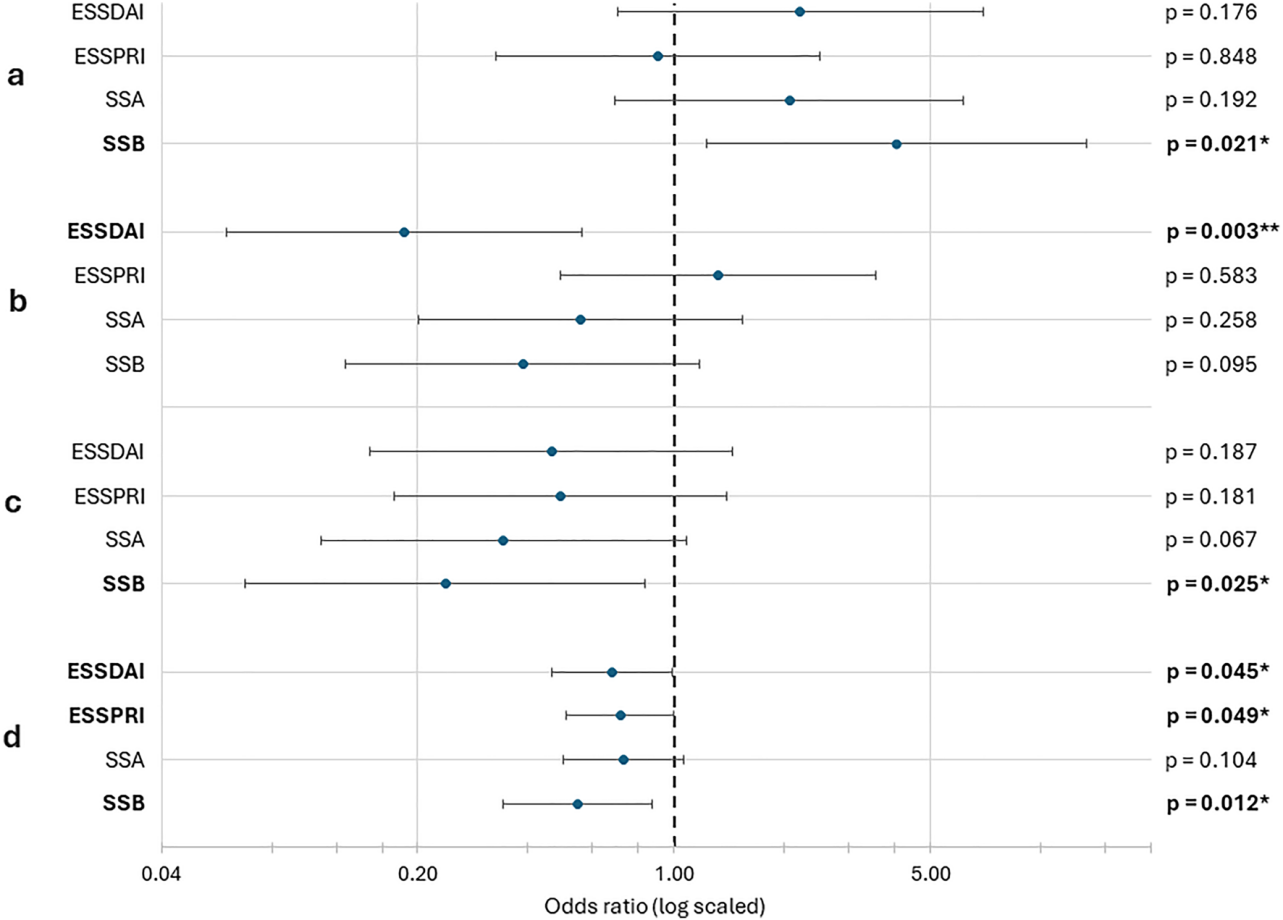
Logistic regression analysis for religiousness (**a**), spirituality (**b**), engagement in (**c**) and duration of individual spiritual activity (**d**). *ESSDAI* EULAR Sjögren’s Syndrome Disease Activity Index, *ESSPRI* EULAR Sjögren’s Syndrome Patient Reported Index, *SSA* anti-Ro/SSA autoantibody, *SSB* anti-La/SSB autoantibody. Confidence interval level of 95% was applied. *p < 0.05; **p < 0.01.

To establish the effect of religiousness, spirituality, engagement in prayer/meditation and its duration on the examined variables, binomial logistic regression analyses were conducted after categorizing the variables as described in the methods section. Increased self-assessed religiousness predicted increased serum level of SSB (OR = 4.048; p = 0.021) (Fig. 2a). Contrarily, higher spirituality values prognosticated lower ESSDAI scores (OR = 0.184; p = 0.003) (Fig. 2b), and engagement in individual spiritual activity predicted lower SSB serum level significantly (OR = 0.238; p = 0.025) and lower SSA level at borderline significance (OR = 0.342; p = 0.067) (Fig. 2c). Aside from that, ESSPRI score and SSA level were not linked to the independent variables significantly.

The analysis of the duration of individual spiritual activity revealed, that the more time the patient spent with this activity, the better outcomes could be measured in the terms of ESSDAI (OR = 0.680; p = 0.045), ESSPRI (OR = 0.714; p = 0.049) and SSB (OR = 0.547; p = 0.012) (Fig. 2d). The same procedure was applied to analyze STS subscales with no significant results (Supplementary Table 6).

## Discussion

Our results indicate that there is a relationship between the examined features; biological measures, such as immunological parameters, self-reported and semi-objective disease activity are related to spiritual engagement and practice in pSS. To our knowledge, this is the first study to assess the relationship between spirituality, immune function and disease activity in pSS. The underlying mechanisms may be related to the mind–body relationship, which is the subject of growing interest in behavioral sciences. The serum level of autoantibodies typical for pSS were decreased when the patient claimed to be spiritual or engaged in prayer/meditation independently from sex, age, disease duration, settlement type, education, living in partnership, smoking, religiousness, spirituality. This association was the strongest in the case of SSA among patients engaged in individual spiritual activity (prayer/meditation). Unexpectedly, these associations were not significant measured with a questionnaire (STS PME). Concerning disease activity markers, perceived skin dryness was the most sensitive symptom regarding the link between spirituality and physical symptoms, followed by perceived tracheal dryness. Decreased ESSDAI score was associated with spiritual attitude and practice in almost every statistical analysis. According to logistic regression analysis, a one-point increase in the level of spirituality made 81,6% less likely, that the patient was going to have clinically detectable disease activity at all.

The evidence presented herein points to a subtle intersection between spirituality and prayer/meditation in particular with immunological properties and hence the health and well-being of the individual. This interplay is presumably the result of the complexity of various psychoneuroimmunological pathways. Stress may be a key player in this phenomenon: while it usually suppresses the immune system, in the case of autoimmune disorders, stress may interfere with the ability of the body to regulate immune functioning, resulting in the excessive or exaggerated responses seen in autoimmune diseases^23^. Since prayer/meditation decreases the stress level, it is very likely that this effect is followed by an increase in the organism’s capacity to regulate its own immune functions. By mitigating stress, spirituality may indirectly support immune function and enhance overall health. Besides its stress-reducing effect, spirituality also enhances positive emotions such as hope, gratitude and compassion. Positive emotions are found to decrease the level of inflammatory markers, such as proinflammatory cytokine interleukin-6 (IL-6)^24^. The decrease of IL-6 and other inflammatory markers has been recognized specifically in the case of religious activity^25^. This may let us conclude that prayer/meditation, as forms of active religious and spiritual practice, has the same effect. This hypothesis is reinforced by findings of anti-inflammatory effects of Buddhist meditation in psoriasis^26^ and Christian intercessory prayer in RA^27^. Contrariwise, proinflammatory cytokines produced by activated immune cells may induce feelings of tiredness, fatigue, and depression^28^. The negative relationship between self-reported fatigue (ESSPRI02) and the duration of individual spiritual activity may be the consequence of this biological pathway.

Moreover, neural correlates of both spirituality and immune mechanisms are worth discussing since nervous tissue could be a link between spirituality and the immune system. Spirituality itself and spiritual activities stimulate the reward system of the brain. This activation is strengthened by sense of purpose in one’s life, which is also a characteristic feature of spiritual mindset^29^. For instance, brain regions associated with representation of reward were reproducibly activated among Mormons while they were ‘feeling the Spirit’. The activation followed by this religious experience was detected within bilateral nucleus accumbens, frontal attentional, and ventromedial prefrontal cortical loci.^30^. Since activation of the reward system boosts both innate and adaptive immunity as well as strengthen immunological host defense via the sympathetic nervous system^31^, it is likely that these experiences lead to better regulation of the immune system and thus to reduced autoimmune mechanisms.

Furthermore, personalized spiritual experiences are described to be related to reduced activity in the left inferior parietal lobule (IPL)^32^. IPL may be responsible for perceptual processing and self-other representations during spiritual experience; thus, the decreased blood supply might indicate loosening of self-other boundaries consistent with the experience of self-transcendence. Its role in pSS is also made likely by the fact that left IPL has been found to be larger among male subjects^33^; which coincides with the gender disequilibrium typical for pSS. In addition, reduced cerebral blood flow was found in the same region related to reduced immune response among patients with undifferentiated somatoform disorder, suggesting that left IPL might have an immunomodulatory role^34^. Consequently, personalized spiritual experience may lead to beneficial immunosuppression in pSS also, which could explain the negative correlation between the duration of individual spiritual activity and ESSDAI.

Besides the semi-objective disease activity score ESSDAI, the subjective measure ESSPRI also showed significant correlations with spiritual variables. The most associations were found in the case of perceived skin dryness (ESSPRI07), which was in a negative relation with engagement in individual spiritual activity, and its duration. The skin, as barrier of the physical body, represents the self and the others on spiritual level. It is the organ of separation and protection, touch and contact, expression and representation, even from maternal-infant bonding with breast feeding. The relationship between the skin and the mind becomes evident when experiencing a strong emotion: dilation or constriction of surface blood vessels and dryness or evaporation from sweating are ‘symptoms’ of different emotions^35^. Hence, the definite relationship between spirituality and skin dryness might be, in a psychosomatic/psychodynamic interpretation the repercussion of loosening the borders of the self during spiritual experience, as described in relation with the decreased blood flow in the left IPL. The other parameter sensitive towards spirituality was tracheal dryness (ESSPRI09). Breathing and breathing techniques have crucial role in spiritual practice. Yoga breathing itself has a stress resilience inducing effect resolving anxious symptoms of psychiatric patients^36^. It is likely that spirituality has a disease modifying effect in the case of respiratory symptoms.

It is important to note, however, that there are some factors related to spirituality and/or religiousness in our study in case of which the relation with disease activity is positive. Among ESSPRI items mental fatigue (ESSPRI04) and oral dryness (ESSPRI06) were in a direct proportion with spirituality. Religiousness was associated with elevated levels of IGG, SSA and SSB. These findings are likely the consequences of negative religious coping. Negative religious coping (also referred to as ‘religious struggle’ or ‘spiritual struggle’) tends to be associated with poorer mental health outcomes^37^. There are three different types of this maladaptive coping mechanism: 1. divine: difficulties or negative feelings towards God or the ‘numinous’; 2. interpersonal: conflicts and judgements regarding other believers; 3. intrapsychic: internal religious guilt and doubt^38^. Since negative religious coping or spiritual struggle is associated with greater distress, the immune function is very likely to worsen in their case due to the opposite mechanism described above regarding stress and immune function^39^. Regarding the result that perceived physical symptoms were more severe mainly in the case of more religious individuals, while spirituality was usually associated with better health outcomes, it is likely that in some cases they antagonize each other. In this concept, religiousness is rather related to the community, meeting its expectations and following its traditions; whilst spirituality represents an inner attitude, the capability to transcend the boundaries of the individual. Hence, the first is likely to enhance, while the second is more likely to reduce stress and maladaptive coping. This may be a relevant finding for the conceptualization of future studies, since religiousness and spirituality are usually examined as one phenomenon, but here we report a study in which they have a different effect on the perceived health of the patients.

The salient negative association between SSA autoantibody and prayer/meditation and its duration and the reduced serum level of SSB autoantibody related to spirituality raises the possibility that spirituality and prayer/meditation may have an immunomodulatory role. This conclusion is supported by the elevated complement component C3 activity related to duration of individual spiritual activity, since it is a molecule responsible for defending the body from outer harms. Thus, it is likely that spirituality and spiritual practice suppresses the autoimmune procedure and enhance the regular immune function. The importance of the duration of prayer/meditation is reinforced by the finding that this variable is associated with decreased level SSA significantly and with SSB level at a borderline significance. Nevertheless SSA and SSB antibody levels are currently not considered as disease activity markers, these results indicate beneficial effect of prayer/meditation duration on pSS. The clinical significance of this result is that the duration of individual spiritual activity can be increased intentionally, which may have a therapeutic value in the terms of physical and mental health.

Besides our findings and conclusions, the introduced research has many limitations. First, it involved a relatively small sample size of 108 patients. This limited sample size might affect the generalizability of the findings to a larger population of individuals with primary Sjögren’s syndrome (pSS). The cross-sectional design of the study does not allow to make conclusions about causality. The assumed causal relationship between spirituality, immune parameters, and disease activity, could also be bidirectional, with disease activity influencing spirituality or engagement in spiritual practices. Furthermore, spirituality may also have an impact on disease activity through other, unstudied factors. For instance, spiritual mindset results in healthier lifestyle choices (e.g. lower incidence of substance abuse^40^, higher sleep quality^41^). Besides, daily spiritual experiences are associated with greater performance of health behaviors, while religious struggle is correlated with less among young cancer survivors, which is coherent with our observations regarding spirituality and religiousness^42^. Hence, lifestyle may be an important mediating factor in the relationship of spirituality and chronic disease activity, which requires further studies. The described biological mechanisms underlying the observed associations are speculative and not directly measured in the study. Such an example is the better functionality of the host immune system mediated by the sympathetic nervous system among spiritual patients. Measurements of autonomic nervous system functions are not involved in the study, although many dysfunctions of it have been described in pSS, such as orthostatic hypotension, postural hypertension, postural tachycardia, thermoregulatory impairment, esophageal, gastric, intestinal dysmotility, genitourinary impairment and respiratory dysmetria^43^. Investigating associations between autonomic functions and spirituality in the future would help us to decide whether this hypothesis of ours is correct.

In summary, we report findings reinforcing that spirituality and prayer/meditation have beneficial effect on immune parameters and disease activity in pSS. Presumably, the mind–body interactions and mechanisms described above are not restricted to pSS only, hence, these observations may be useful to understand the interactions of spirituality and physical health and mind–body relationship. The study underlines the disease modifying effect of spirituality implying that holistic mental health care is important in the treatment of pSS and chronic disorders in general. The scientific and clinical application of these findings may hopefully contribute to paving the way towards an authentic BPSS model of health and disease.

## Conclusions

Spirituality correlates with immune parameters and disease activity in primary Sjögren’s syndrome. Patients with spiritual attitude are less likely to have increased disease activity. Besides being spiritual, engagement in individual spiritual activities, such as prayer/meditation has beneficial disease modifying effect. However, it is important to make a distinction between religiousness and spirituality based on our results, since religiousness may worsen the disease course. contrary to spirituality. These changes are supposedly due to psychoneuroimmunological pathways. In addition to the biologically measurable variables, the alleviation and aggravation of perceived symptoms (pain, dryness, fatigue) are important outcomes of spiritual engagement and practice.

## Methods

### Sample

Patient recruitment for the study was took place at the Autoimmune Sjögren specialty clinic, Division of Clinical Immunology, Institute of Internal Medicine, Clinical Centre, University of Debrecen, involving 112 patients. The inclusion criteria were the primary nature of the disease, the absence of other systemic autoimmune disorders and intact cognitive functions.

### Clinical data and assessment of spirituality

The following immunological parameters of the patients were recorded: neutrophil granulocyte count (Neu), lymphocyte count (Ly), hemoglobin concentration (HGB), platelet count (PLT), complement component C3, C4 and total complement activity (C3, C4 and CH50 respectively), and serum levels of immunoglobulin G (IgG), rheumatoid factor (RF), anti-Ro/SSA (SSA) and anti-La/SSB (SSB) autoantibodies. EULAR Sjögren’s Syndrome Disease Activity Index (ESSDAI) score was calculated for each patient^44^. To assess perceived disease activity, participants completed the EULAR Sjögren’s Syndrome Patient Reported Index (ESSPRI)^45^, of which the final score and individual items were analyzed separately. Spirituality and religiousness were assessed with two methods. First, direct questions were asked:

- (1) ‘How would you assess the extent of your own religiousness?’ (Possible answers on a 4-point Likert-scale: very religious, religious, little religious, not religious);
- (2) ‘How would you assess the extent of your own spirituality?’ (Possible answers on a 4-point Likert-scale: very spiritual, spiritual, little spiritual, non-spiritual);
- (3) ‘Do you engage in any individual activities that are not related to organized religion and that have a religious/spiritual role in your life (e.g. prayer, meditation)?’ (Possible answers: yes/no) (note: later referred to as engagement in individual spiritual activity or prayer/meditation)
- (4) ‘If your answer to the previous question is yes, how many hours in an average week do you spend on that activity?’.

Second, we applied the Spiritual Transcendence Scale, a 24-item questionnaire containing originally three subscales: Universality, Prayer Fulfillment, and Connectedness^22^. During data processing we applied a new subscale structure, that was later suggested by a study based on exploratory factor analysis, resulting in 5 subscales: Prayer/Meditation Enjoyment (PME), Universal Connectedness (UC), Greater Purpose (GP), Wholeness of Humanity (WH) and Closeness to the Deceased (CD)^46^.

### Statistical analyses

After data collection, statistical analysis was performed, during which 4 patients got excluded from the study for incomplete answers. The statistical analysis was conducted using SPSS (version 26) software (IBM Company, Armonk, NY, USA). Prevalence data were compared by the χ2 test. First, we compared immune parameters and disease activity measures between non-spiritual and spiritual patients (Table 2). The same analysis was applied between little or not religious and religious or very religious groups with less significant results (see Supplementary Table 2). At grouping of our sample by spirituality we put patients reporting themselves non-spiritual in one group and the patients belonging to the three other categories were labeled as spiritual. At religiousness, however, we considered not or little religious patients as one category and religious and very religious as the other one. The reason for this distinction is that we considered spirituality as a nominal variable, as an attitude that can be there or not, whilst religiousness was regarded as an ordinal variable depending on the level of commitment to traditions, customs and community norms. Along these lines, the non-spiritual group consisted of 48, while the spiritual group consisted of 60 participants. Comparisons between groups were performed by Student’s unpaired t-test in case of normally distributed variables and by Mann–Whitney U-test in case of variables with non-normal distribution. Shapiro–Wilk test was used to examine whether the quantitative variables were normally distributed or not, and, if necessary, Templeton’s two-step method was considered to transform the non-normal variables into normal ones^47^. This was followed by a linear regression analysis, that examined the effect of spirituality, engagement in prayer/meditation and the duration of it on immune and disease activity parameters. Moreover, after establishing two categories for each of the four most decisive markers of disease activity (ESSDAI, ESSPRI, SSA, SSB) binary logistic regression analysis was performed. The categories were ESSDAI = 0 (no disease activity), ESSDAI >  = 1 (detectable disease activity) for ESSDAI; ESSPRI score = 0–4 (none or mild perceived disease activity), ESSPRI score = 5–10 (moderate or severe perceived disease activity) for ESSPRI; and in the case of SSA and SSB the distinction point was 10 U/ml (10 U/ml or lower: not significant increase; above 10 U/ml: clinically significant increase). All regression analyses were conducted using a model adjusted for relevant factors (sex, age, disease duration, settlement type, education, living in partnership, smoking, religiousness, spirituality, and engagement in prayer/meditation).

### Ethical approval and informed consent

The study was conducted according to the guidelines of the Declaration of Helsinki and approved by the Institutional Ethics Committee of the University of Debrecen (protocol code: 5614-2020, date of approval: 17.12.2020). Informed consent was obtained from all subjects involved in the study.

### Supplementary Information

Supplementary Tables.

## Data Availability

Data available on request from the authors.
