# Supplementary material for: Spirituality is associated with immune parameters and disease activity in primary Sjögren’s syndrome: a cross-sectional study
Source: Sci Rep. 2024 May 30;14:12473. doi: 10.1038/s41598-024-62801-w (PMC11139944; doi:10.1038/s41598-024-62801-w)
Supplement: Supplementary file 1 — Supplementary Tables. [file 41598_2024_62801_MOESM1_ESM.pdf]

Supplementary information

		Not or little religious (n = 55)	Religious or very religious (n = 53)	p-value
		Average (95%CI)		
Age (year)		59.45 (56.34 – 62.57)	59.64 (56.05 – 63.23)	0.770
Disease duration		16.61 (13.85 – 19.37)	14.06 (11.97 – 16.15)	0.390
Duration of prayer/meditation (hours/week)		0.31 (0.00 – 0.69)	1.30 (0.72 – 1.89)	<0.001**
		Prevalence in % (95% CI)		p-value
Female		85.45 (74.42 – 92.88)	94.34 (85.66 – 98.38)	0.127
Settlement type	Capital or city	47.27 (34.50 – 60.32)	28.30 (17.56 – 41.34)	0.052
	Small town	36.36 (24.61 – 49.51)	37.74 (25.62 – 51.15)	
	Rural	16.36 (8.42 – 27.74)	33.96 (22.33 – 47.29)	
Education	Not completed or completed primary school	10.91 (4.68 – 21.11)	5.66 (1.62 – 14.34)	0.590
	High school	50.91 (37.94 – 63.79)	56.60 (43.21 – 69.30)	
	College/university diploma	38.18 (26.21 – 51.36)	37.74 (25.62 – 51.15)	
Living in partnership		61.82 (48.64 – 73.79)	67.92 (54.67 – 79.28)	0.506
Smokers		7.27 82.50 – 16.37)	3.77 (0.79 – 11.56)	0.427
Engaged in individual spiritual activity		12.73 (5.88 – 23.37)	56.60 (43.21 – 69.30)	<0.001**
Self-assessed level of spirituality	Non-spiritual	63.64 (50.49 – 75.39)	24.53 (14.50 – 37.25)	<0.001**
	Little spiritual	25.45 (15.40 – 38.01)	18.87 (10.14 – 30.89)	
	Very spiritual or spiritual	10.91 (4.68 – 21.11)	56.60 (43.21 – 69.30)	

*: p <0.05; **: p<0.01

Supplementary table 1. Comparison of sociodemographic characteristics and self-assessed measures of spirituality and religiousness of the research sample between not or little religious and religious or very religious groups. CI: confidence interval. Confidence interval level of 95% was applied.

	Non-spiritual (n = 48)	Spiritual (n = 60)	p-value
	Average (95%CI)		
NEU	4.05 (3.59 – 4.51)	3.77 (3.35 – 4.19)	0.264
LY	1.44 (1.26 – 1.62)	1.61 (1.44 – 1.78)	0.124
HGB	133.96 (130.69 – 137.23)	137.87 (134.54 – 141.19)	0.201
PLT	227.30 (211.65 – 242.95)	232.12 (219.24 – 244.99)	0.629
C3	1.28 (1.21 - 1.35)	1.26 (1.19 - 1.33)	0.829
C4	0.23 (0.21 - 0.25)	0.25 (0.20 - 0.30)	0.792
CH50	69.50 (61.79 - 77.21)	71.90 (65.78 - 78.02)	0.555

Supplementary table 2. Comparison of blood cell counts and complement activities between non-spiritual and spiritual groups. NEU: neutrophil granulocyte count, LY: lymphocyte count, HGB: hemoglobin concentration, PLT: platelet count, C3, C4, CH50: complement component C3, C4 and total complement activity respectively, CI: confidence interval. Confidence interval level of 95% was applied.

	Not or little religious (n = 55)	Religious or very religious (n = 53)	p-value
	Average (95%CI)		
NEU	3.93 (3.52 - 4.34)	3.85 (3.39 - 4.32)	0.625
LY	1.39 (1.25 - 1.54)	1.69 (1.49 - 1.88)	0.019*
HGB	137.36 134 (20 -140.53)	134.87 (131.31 - 138.42)	0.159
PLT	224.53 (210.76 - 238.30)	235.79 (221.50 - 250.08)	0.198
C3	1.26 (1.20 - 1.31)	1.28 (1.20 - 1.36)	0.591
C4	0.22 (0.20 - 0.24)	0.26 (0.21 - 0.32)	0.293
CH50	69.31 (62.97 -75.65)	72.42 (65.12 - 79.71)	0.561
IGG	13.06 (11.67 - 14.44)	16.04 (13.33 - 18.75)	0.136
RF	32.24 (16.99 - 47.48)	46.34 (13.71 - 78.97)	0.404
SSA	41.62 (32.33 - 50.90)	47.34 (37.34 - 57.34)	0.278
SSB	26.60 (18.54 - 34.66)	36.06 (26.06 - 46.05)	0.110
ESSDAI	4.80 (4.10 - 5.49)	4.80 (4.10 - 5.49)	0.404
ESSPRI score	4.80 (4.10 - 5.49)	4.80 (4.10 - 5.49)	0.156
ESSPRI main items (1-3)			
dryness (ESSPRI01)	5.89 (5.33 - 6.45)	4.81 (4.13 - 5.49)	0.035*
fatigue (ESSPRI02)	5.69 (4.99 - 6.39)	4.81 (3.99 - 5.62)	0.102
limb pain (ESSPRI03)	4.95 (4.08 - 5.81)	5.02 (4.16 - 5.87)	0.992
ESSPRI items 4-10			
mental fatigue (ESSPRI04)	2.98 (2.35 - 3.62)	2.92 (2.19 - 3.65)	0.803
ESSPRI dryness items			
ocular (ESSPRI05)	5.67 (5.03 - 6.31)	4.57 (3.83 - 5.31)	0.026
oral (ESSPRI06)	5.55 (4.76 - 6.34)	4.94 (4.21 - 5.68)	0.214
skin (ESSPRI07)	5.27 (4.60 - 5.95)	4.33 (3.57 - 5.10)	0.062
nasal (ESSPRI08)	3.83 (3.02 - 4.65)	3.35 (2.58 - 4.12)	0.400
tracheal (ESSPRI09)	4.00 (3.25 - 4.75)	3.71 (2.82 - 4.59)	0.577
vaginal (ESSPRI10)	2.92 (1.99 - 3.85)	2.57 (1.80 - 3.34)	0.926

*: p <0.05; **: p<0.01

Supplementary table 3. Comparison of blood cell counts, immune parameters and disease activity markers between not or little religious and religious or very religious groups. NEU: neutrophil granulocyte count, LY: lymphocyte count, HGB: hemoglobin concentration, PLT: platelet count, C3, C4, CH50: complement component C3, C4 and total complement activity respectively, IGG: Immunoglobulin G, RF: rheumatoid factor, SSA: anti-Ro/SSA autoantibody, SSB: anti-La/SSB autoantibody, ESSDAI: EULAR Sjögren's Syndrome Disease Activity Index, ESSPRI: EULAR Sjögren's Syndrome Patient Reported Index, CI: confidence interval. Confidence interval level of 95% was applied.

	Religiousness	
	B-value (Std. err.)	p-value
IGG	3.732 (1.839)	0.045*
RF	25.717 (17.926)	0.155
SSA	15.731 (6.996)	0.027*
SSB	14.744 (5.773)	0.012*
ESSDAI	0.246 (0.752)	0.744
ESSPRI score	-3.62 (0525)	0.492
ESSPRI main items (1-3)		
dryness (ESSPRI01)	-0.785 (0.533)	0.144
fatigue (ESSPRI02)	-0.267 (0.635)	0.675
limb pain (ESSPRI03)	0.002 (0.661)	0.997
ESSPRI items 4-10		
mental fatigue (ESSPRI04)	-0.790 (0.546)	0.151
ESSPRI dryness items		
ocular (ESSPRI05)	-0.662 (0.594)	0.268
oral (ESSPRI06)	-0.598 (0.659)	0.366
skin (ESSPRI07)	-0.034 (0.576)	0.953
nasal (ESSPRI08)	-0.329 (0.659)	0.619
tracheal (ESSPRI09)	0.448 (0.639)	0.485
vaginal (ESSPRI10)	0.075 (0.665)	0.911

*: p <0.05; **: p<0.01

Supplementary table 4. Linear regression of religiousness adjusted for sex, age, disease duration, type of the settlement, education, living in partnership, smoking, spirituality, engagement in prayer/meditation. IGG: Immunoglobulin G, RF: rheumatoid factor, SSA: anti-Ro/SSA autoantibody, SSB: anti-La/SSB autoantibody, ESSDAI: EULAR Sjögren's Syndrome Disease Activity Index, ESSPRI: EULAR Sjögren's Syndrome Patient Reported Index. Standard error ranges for B values in parentheses.

	Prayer/Meditation Enjoyment		Universal Connectedness		Greater Purpose		Wholeness of Humanity		Closeness to the Deceased	
	B-value (Std. err.)	p-value	B-value (Std. err.)	p-value	B-value (Std. err.)	p-value	B-value (Std. err.)	p-value	B-value (Std. err.)	p-value
NEU	0.016 (0.031)	0.602	-0.015 (0.040)	0.717	0.054 (0.068)	0.426	0.046 (0.046)	0.317	-0.029 (0.078)	0.708
LY	0.018 (0.012)	0.154	0.000 (0.016)	0.982	-0.023 (0.027)	0.400	-0.029 (0.018)	0.112	0.037 (0.031)	0.241
HGB	0.157 (0.224)	0.487	0.654 (0.294)	0.231	-0.366 (0.492)	0.459	-0.095 (0.336)	0.778	0.825 (0.558)	0.143
PLT	0.799 (0.973)	0.414	2.209 (1.269)	0.085	1.227 (2.153)	0.570	1.856 (1.451)	0.204	2.460 (2.447)	0.317
C3	-0.002 (0.005)	0.655	-0.020 (0.007)	0.003**	-0.026 (0.011)	0.026*	-0.005 (0.008)	0.562	-0.022 (0.013)	0.088
C4	0.003 (0.003)	0.212	-0.005 (0.004)	0.198	-0.003 (0.006)	0.587	0.003 (0.004)	0.416	-0.008 (0.007)	0.240
CH50	0.106 (0.499)	0.833	-0.038 (0.668)	0.954	-0.176 (1.098)	0.873	-0.546 (0.750)	0.469	-0.469 (1.250)	0.708
IGG	-0.233 (0.151)	0.126	0.010 (0.203)	0.963	0.559 (0.331)	0.095	0.241 (0.229)	0.296	-0.364 (0.384)	0.345
RF	-0.987 (1.502)	0.517	-1.023 (1.982)	0.607	1.354 (3.280)	0.681	1.523 (2.255)	0.501	-1.374 (3.733)	0.714
SSA	-0.812 (0.576)	0.162	-0.382 (0.777)	0.624	0.687 (1.280)	0.593	0.385 (0.881)	0.663	-0.346 (1.464)	0.814
SSB	-0.148 (0.480)	0.759	0.002 (0.624)	0.997	1.481 (1.047)	0.160	0.769 (0.723)	0.290	-0.811 (1.205)	0.503
ESSDAI	-0.055 (0.064)	0.389	-0.042 (0.085)	0.622	0.027 (0.140)	0.846	0.137 (0.097)	0.159	-0.151 (0.156)	0.336
ESSPRI score	-0.34 (0.043)	0.442	-0.022 (0.060)	0.714	-0.105 (0.096)	0.277	-0.051 (0.066)	0.439	0.150 (0.110)	0.343
ESSPRI main items										
dryness (ESSPRI01)	0.000 (0.015)	0.999	-0.013 (0.059)	0.827	-0.090 (0.097)	0.359	-0.010 (0.068)	0.886	0.053 (0.113)	0.640
fatigue (ESSPRI02)	0.037 (0.053)	0.491	0.044 (0.071)	0.536	0.028 (0.116)	0.813	0.053 (0.080)	0.509	0.197 (0.133)	0.143
limb pain (ESSPRI03)	-0.029 (0.056)	0.602	-0.023 (0.074)	0.750	-0.188 (0.120)	0.119	-0.118 (0.083)	0.156	0.135 (0.140)	0.335
ESSPRI items 4-10										
mental fatigue (ESSPRI04)	0.065 (0.045)	0.156	0.073 (0.060)	0.231	0.038 (0.099)	0.701	-0.020 (0.068)	0.773	0.276 (0.110)	0.014*
ESSPRI dryness items										
ocular (ESSPRI05)	0.065 (0.049)	0.193	0.056 (0.066)	0.399	-0.042 (0.108)	0.698	-0.006 (0.074)	0.939	-0.099 (0.123)	0.423
oral (ESSPRI06)	-0.020 (0.055)	0.719	-0.058 (0.073)	0.425	-0.149 (0.119)	0.213	-0.026 (0.082)	0.753	0.115 (0.137)	0.402
skin (ESSPRI07)	-0.062 (0.048)	0.197	-0.022 (0.064)	0.729	-0.031 (0.105)	0.765	-0.081 (0.071)	0.255	0.070 (0.120)	0.561
nasal (ESSPRI08)	-0.029 (0.055)	0.604	-0.024 (0.073)	0.747	-0.012 (0.121)	0.922	-0.084 (0.081)	0.304	0.059 (0.137)	0.667

tracheal (ESSPRI09)	-0.059 (0.053)	0.265	-0.007 (0.071)	0.917	0.017 (0.117)	0.885	-0.046 (0.079)	0.561	0.157 (0.131)	0.235
vaginal (ESSPRI10)	-0.118 (0.056)	0.038*	-0.017 (0.078)	0.832	-0.056 (0.121)	0.62	0.011 (0.087)	0.904	-0.019 (0.134)	0.886

*: p <0.05; **: p<0.01

Supplementary table 5. Linear regression adjusted for sex, age, disease duration, type of the settlement, education, living in partnership, smoking, religiousness, spirituality, engagement in prayer/meditation and one of the five subscales of the Spiritual Transcendence Scale. NEU: neutrophil granulocyte count, LY: lymphocyte count, HGB: hemoglobin concentration, PLT: platelet count, C3, C4, CH50: complement component C3, C4 and total complement activity respectively, IGG: Immunoglobulin G, RF: rheumatoid factor, SSA: anti-Ro/SSA autoantibody, SSB: anti-La/SSB autoantibody, ESSDAI: EULAR Sjögren's Syndrome Disease Activity Index, ESSPRI: EULAR Sjögren's Syndrome Patient Reported Index. Standard error ranges of B values in parentheses.

	Prayer/Meditation Enjoyment		Universal Connectedness		Greater Purpose		Wholeness of Humanity		Closeness to the Deceased	
	OR (95%CI)	p-value	OR (95%CI)	p-value	OR (95%CI)	p-value	OR (95%CI)	p-value	OR (95%CI)	p-value
ESSDAI	0.950 (0.864- 1.045)	0.295	0.912 (0.807 – 1.030)	0.137	0.985 (0.807 – 1.201)	0.878	1.026 (0.892 – 1.181)	0.717	0.920 (0.729 – 1.160)	0.479
ESSPRI	0.982 (0.902 – 1-068)	0.667	1.006 (0.897 – 1.128)	0.921	0.917 (0.758 – 1.109)	0.371	0.951 (0.835 – 1.082)	0.444	1.187 (0.950 – 1.482)	0.131
SSA	0.933 (0.848 – 1.027)	0.156	1.019 (0.906 – 1.145)	0.755	1.061 (0.873 – 1.290)	0.552	1.131 (0.982 – 1.301)	0.087	0.900 (0.715 – 1.134)	0.373
SSB	0.951 (0.861 – 1.049)	0.315	0.990 (0.871 – 1.125)	0.876	1.117 (0.897 – 1.392)	0.323	1.119 (0.957 – 1.308)	0.157	0.848 (0.653 – 1.100)	0.848

*: p <0.05; **: p<0.01

Supplementary table 6. Logistic regression analysis for the five subscales of the Spiritual Transcendence Scale. ESSDAI: EULAR Sjögren's Syndrome Disease Activity Index, ESSPRI: EULAR Sjögren's Syndrome Patient Reported Index, SSA: anti-Ro/SSA autoantibody, SSB: anti-La/SSB autoantibody, OR: odds ratio, CI: confidence interval. Confidence interval level of 95% was applied.
